# A Community Detection Model Based on Dynamic Propagation-Aware Multi-Hop Feature Aggregation

**DOI:** 10.3390/e27101053

**Published:** 2025-10-10

**Authors:** Chao Lei, Yuzhi Xiao, Sheng Jin, Tao Huang, Chuang Zhang, Meng Cheng

**Affiliations:** 1 School of Computer Science, Qinghai Normal University, Xining 810008, China; ndust1853764@gmail.com (C.L.); taohuang658@gmail.com (T.H.);; 2 The State Key Laboratory of Tibetan Intelligence, Qinghai Normal University, Xining 810008, China

**Keywords:** community detection, dynamic propagation modeling, adaptive graph sampling, multi-hop feature aggregation, gated mechanism

## Abstract

Community detection is a crucial technique for uncovering latent network structures, analyzing group behaviors, and understanding information dissemination pathways. Existing methods predominantly rely on static graph structural features, while neglecting the intrinsic dynamic patterns of information diffusion and nonlinear attenuation within static networks. To address these limitations, we propose DAMA, a community detection model that integrates dynamic propagation-aware feature modeling with adaptive multi-hop structural aggregation. First, an Information Flow Matrix (IFM) is constructed to quantify the nonlinear attenuation of information propagation between nodes, thereby enriching static structural representations with nonlinear propagation dynamics. Second, we propose an Adaptive Sparse Sampling Module that adaptively retains influential neighbors by applying multi-level propagation thresholds, improving structural denoising and preserving essential diffusion pathways. Finally, we design a Hierarchical Multi-Hop Aggregation Framework, which employs a dual-gating mechanism to adaptively integrate neighborhood representations across multiple hops. This approach enables more expressive structural embeddings by progressively combining local and extended topological information. Experimental results demonstrate that DAMA achieves better performance in community detection tasks across multiple real-world networks and LFR-generated synthetic networks.

## 1. Introduction

With the exponential growth of complex networks, inter-node interaction patterns exhibit dynamic and evolving characteristics. However, existing network representation frameworks still primarily rely on static graph models for structural abstraction [1]. As a core technique for uncovering functional modules, predicting information dissemination pathways, and identifying pivotal hubs, community detection holds significant value in complex systems with complex interaction patterns and modular structures, such as social networks and biological protein interaction networks [2,3]. These networks frequently exhibit hierarchical and multi-scale structures, where accurately identifying community boundaries is essential to uncovering their underlying organizational principles [4,5]. However, many real-world networks are not only structurally complex but also involve dynamic information flows, which pose new challenges for community detection [6]. Current community detection methods face limitations in characterizing information dissemination processes within real-world networks.

Modularity optimization approaches identify community structures by maximizing the disparity between internal connectivity densities and those expected in random networks [7]. Nevertheless, a critical limitation arises: since these methods rely on static structures, they struggle to capture the nonlinear attenuation patterns in multi-hop information propagation. For instance, in social networks, the influence of topics diminishes with increasing propagation distance, while in protein interaction networks, signal transduction efficiency is constrained by topological distance. Although differing in nature, both exemplify the hierarchical and nonlinear decay of information dissemination [8]. Furthermore, modularity-based approaches tend to prioritize local structural metrics, which may overlook inter-community dependencies and lead to suboptimal global community structures. For example, they are prone to misjudgments in ring-shaped graphs or networks with bridging nodes [9,10]. Although recent multi-objective optimization-based community detection methods attempt to balance internal cohesion maximization and inter-community linkage minimization, they remain predominantly reliant on static topological information. As a result, they lack the capacity to model node-level information coupling and heterogeneous propagation patterns [11,12].

The stochastic block model (SBM), as another classical community detection framework, assumes identical or similar connection probabilities among nodes within the same community. This rigid homogeneity assumption struggles to accommodate the heterogeneity of node connection patterns observed in real-world networks [13]. such as the “core-periphery” structure in social networks or interdisciplinary connections in academic networks [14]. While enhanced versions like the degree-corrected SBM (DC-SBM) mitigate this issue to some extent, they still fail to capture the temporal dynamics and dynamic coupling characteristics inherent in information dissemination processes [15,16].

Graph neural networks (GNNs) introduce a novel paradigm for community detection tasks, leveraging graph convolutional or attention mechanisms to recursively aggregate information from multi-hop neighborhoods [17]. However, most existing GNNs adopt fixed-depth architectures, which constrain the receptive field and limit the ability to capture complex propagation patterns and semantic dependencies between distantly connected nodes [18,19].

While a variety of community detection methods have achieved notable progress, most existing approaches still face challenges in capturing nonlinear, long-range, and dynamic-like propagation patterns that characterize many real-world networks. These limitations highlight the potential benefits of developing models that, even when operating on static topologies, can approximate certain aspects of temporal diffusion, such as long-range interactions and propagation attenuation. To this end, we introduce a Dynamic Propagation-Aware Multi-Hop Aggregation Model (DAMA). Although DAMA is built for static graphs, it incorporates propagation-aware mechanisms—such as the IFM—derived solely from static topological features, without relying on time-evolving snapshots. In doing so, DAMA approximates diffusion-like behaviors in a static setting, aiming to bridge the gap between structural topology and dynamic information flow. The primary contributions of this work are summarized as follows:

1. Information Flow Matrix (IFM): We construct an IFM to model nonlinear attenuation in node-to-node propagation strength within static graphs. This matrix helps the model to better account for multi-step propagation, aiding in the detection of dependencies that may be weakened or overlooked in traditional adjacency-based models. Such capability is essential for effectively modeling complex propagation patterns in static networks.
2. Adaptive Sparse Sampling: We propose an adaptive sampling mechanism that selectively retains neighbors with high propagation strength based on dynamically adjusted hierarchical thresholds. This reduces redundant connections and suppresses noise within the neighborhood structure, enhancing structural clarity.
3. Hierarchical Multi-Hop Gated Aggregation: We design a hierarchical multi-hop aggregation mechanism with a dual-gating strategy. By combining node-level propagation intensity with batch-wise feature statistics, the model adaptively balances the contributions of different hop-level features, enabling flexible modeling of hierarchical dependencies in static networks.

## 2. Related Work

A broad range of community detection methods have been developed to analyze structural patterns in complex networks. Traditional approaches include modularity optimization, probabilistic generative models such as the stochastic block model (SBM), and, more recently, graph neural networks (GNNs). While primarily designed for static graphs, recent efforts have sought to incorporate temporal or propagation-aware cues into these models. For instance, degree-corrected SBM variants account for local structural heterogeneity, while dynamic GNNs leverage graph snapshots to model temporal transitions. However, a persistent challenge remains: effectively modeling long-range dependencies and attenuation in information propagation using only static topological structures.

Modularity Optimization-based Methods: Modularity-driven community detection represents a classical paradigm. Greedy modularity optimization algorithms, such as Louvain, are widely adopted for their simplicity but are often trapped in local optima [20]. Rustamaji et al. propose an improved method based on modularity decomposition, which significantly enhances modularity through an innovative node-community disassembly strategy [21]. Yuan et al. introduced the Modularity Subset Maximization (MSM) algorithm, transforming modularity maximization into a non-convex subset identification problem solved via difference-of-convex programming [22]. Despite their effectiveness on static networks, these methods suffer from resolution limits, local optima, and neglect of node attributes, hindering their ability to capture hierarchical network structures [23].

Probabilistic Generative Models: Stochastic block models (SBMs) characterize community structures by defining probabilistic network generation processes. Classical variants, including mixed-membership SBM (MMSB) and degree-corrected SBM (DCSBM), have been extensively studied to model overlapping communities and degree heterogeneity [24,25]. Sun et al. propose the vGraph framework, a probabilistic generative model for joint node-community representation learning, enabling both overlapping and non-overlapping community detection [26]. However, such models typically assume static and simplistic network structures, inadequately supporting complex multi-hop topological relationships, dynamic evolution, and heterogeneous node attributes [27,28].

Graph Neural Network-based Methods: Deep learning approaches leveraging GNNs have recently emerged as a research hotspot. Sobolevsky et al. developed a recurrent GNN variant for unsupervised community detection via modularity optimization, enabling continuous optimization of partitioning quality functions [29]. Zhou et al. proposed the Bernoulli–Poisson Graph Convolutional Network (BP-GCN) for heterogeneous social networks, which integrates self-attention mechanisms to identify node relations on symmetric contextual paths and achieves end-to-end community detection [30]. Li et al. propose a dynamic graph community detection algorithm that combines graph convolutional networks with contrastive learning, capturing temporal evolution through relevance aggregation and feature smoothing [31]. Although GNN-based methods excel on large-scale graphs, most existing models assume homogeneous networks or fixed topologies, limiting their capability to model heterogeneous propagation and multi-scale structures. Compared with dynamic graph neural networks such as ST-GCN, which operate on time-ordered graph sequences, DAMA adopts a static-topology-aware dynamic modeling strategy. It encodes information decay through an IFM derived from structural proximity and potential propagation bias. This avoids the need for temporal snapshots while still capturing multi-hop and hierarchical propagation patterns, approximating diffusion intensity through topological proximity and distance-based attenuation. In contrast, GraphSAGE performs fixed-step neighborhood sampling without modeling the strength or decay of information across distances [32]. Furthermore, Dynamic GNNs such as DySAT and EvolveGCN are designed to handle evolving graph structures. DySAT captures both structural and temporal dependencies through self-attention layers, while EvolveGCN models GCN weight dynamics via recurrent networks [33,34]. These models typically rely on sequential graph snapshots, which may not be available in static settings. In contrast, DAMA is designed to operate entirely on static graphs. It captures dynamic-like propagation behavior through potential-based influence matrices derived from static topology, rather than learning from time-variant edge or node dynamics. Our approach provides a static-graph alternative that approximates dynamic propagation without requiring temporal.

Other Classical Approaches: Label propagation algorithms (LPA) are renowned for their simplicity and efficiency. Li et al. enhanced LPA with modularity optimization and node importance (LPA-MNI), reducing randomness by initial community identification followed by label updating based on node importance [35]. Spectral clustering partitions nodes via Laplacian matrix eigenvectors but requires predefined community numbers and incurs high computational costs, making it unsuitable for extremely large networks. For multi-modal networks, multi-view learning has been explored. For instance, Lin et al. proposed Multi-view Attributed Graph Clustering (MvAGC), which integrates attribute perspectives via graph filtering and anchor selection to improve community partitioning [36]. Although effective in certain scenarios, these methods still face inherent limitations. Existing community detection methods have achieved notable success on static networks but face persistent challenges in handling dynamic evolution, nonlinear propagation attenuation, and heterogeneous interaction patterns.

In summary, while existing methods contribute significantly to static graph modeling, they often lack mechanisms for simulating dynamic propagation over static structures. To address this, we propose DAMA, which bridges this gap by embedding diffusion-like behaviors into static topologies through influence-aware multi-hop aggregation, introducing dynamic-like propagation modeling on static graphs via influence-based matrices and adaptive multi-hop feature fusion.

## 3. DAMA: Model Architecture

**Figure 1:**
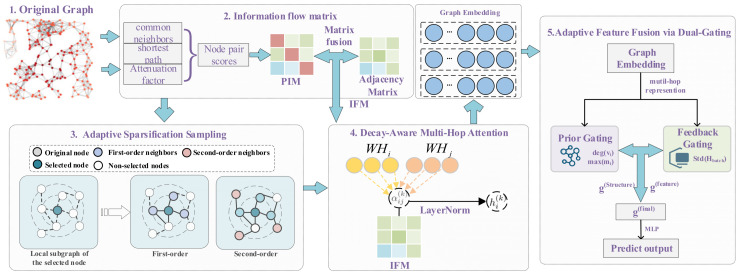
The architecture of the proposed DAMA model.

The overall architecture of the proposed DAMA model is shown in Figure 1. It comprises three interlinked components: an IFM that captures topological propagation influence within static graphs, an adaptive sparse sampling module that constructs hierarchical subgraphs by retaining high-influence neighbors based on IFM weights, and a hierarchical gated aggregation mechanism that dynamically fuses multi-hop features to generate community-sensitive node representations. The following subsections elaborate on the design and implementation of each component.

### 3.1. Information Flow Matrix Construction

**Figure 2:**
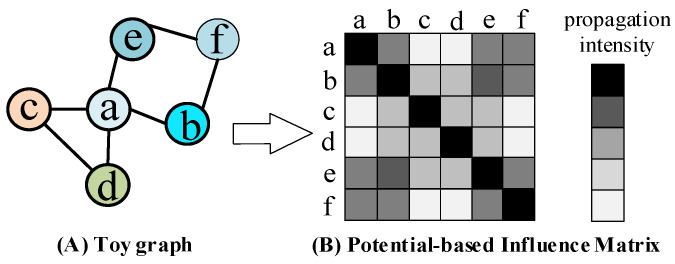
Structure of the Potential-based Influence Matrix (PIM), where higher intensity (darker color) denotes stronger potential influence.

The Potential-based Influence Matrix (PIM), denoted as $P\in {\mathbb{R}}^{n\times n}$, quantifies the strength of information propagation between node pairs in a graph. An example of its matrix structure is illustrated in Figure 2.

Inspired by epidemic diffusion models such as the Susceptible-Infected (SI) model, as well as principles from wireless communications, PIM is based on the intuition that information tends to flow from nodes with higher potential to those with lower potential, with signal strength attenuating over distance. Here, the “potential” reflects the node’s propensity to propagate information, influenced by its local connectivity.

To model the attenuation effect, PIM employs an exponential decay function $e^{-\lambda d_{ij}}$, where $d_{ij}$ denotes the shortest path length between nodes $v_{i}$ and $v_{j}$, and λ controls the decay rate. This function captures the natural weakening of influence as information travels further through the network.

Besides distance, PIM incorporates neighborhood overlap to reflect structural similarity; nodes sharing more common neighbors are more likely to influence each other. The influence score between nodes $v_{i}$ and $v_{j}$ is thus defined as

(1) $$p_{ij}=\left|\Gamma (v_{i})\cap \Gamma (v_{j})\right|\cdot e^{-\lambda d_{ij}}$$

where $\Gamma (v_{i})$ is the set of neighbors of $v_{i}$. This formulation models a potential-driven spatial diffusion process that primarily emphasizes short-range proximity and local structural similarity.

Notably, the importance of first- and second-order neighborhoods has been highlighted in prior work; for example, Ran et al. systematically incorporated micro- (node-pair) and mesoscopic (community-level) structural features into a machine learning framework for community detection, demonstrating that such low-order structural dependencies are particularly informative [37]. Our PIM formulation is consistent with this principle, as it jointly leverages distance decay and local neighborhood overlap to capture influence between nodes.

**Figure 3:**
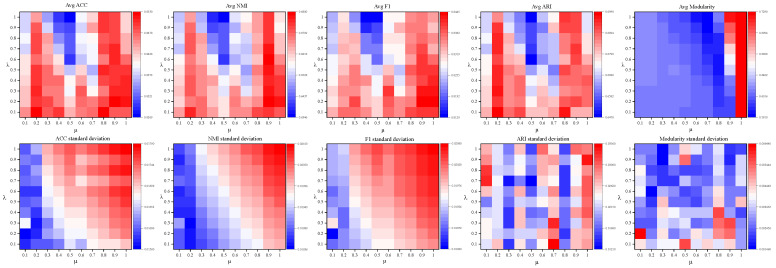
Heatmaps of Mean Values and Standard Deviations for Accuracy, NMI, and F1 Metrics.

Empirical studies indicate that when $d_{ij}>3$, the decay reduces the influence below 50%, consistent with the “three degrees of influence” theory in social networks [38,39]. As detailed in Section 4.4 (Figure 3), hyperparameter tuning shows that smaller λ values enhance model stability. Therefore, we set λ=0.3 to balance empirical robustness and theoretical consistency.

To combine propagation dynamics and graph topology, we define an IFM as a weighted sum of the PIM and the adjacency matrix A:

(2) IFM=βP+γA

Based on the results reported in Appendix A.1 Table A1, Table A2, Table A3, Table A4, Table A5, Table A6 and Table A7, we select the hyperparameter combinations that achieve the best overall performance for each dataset. This configuration balances structural locality and diffusion influence, favoring short-range information while retaining sensitivity to potential longer-range influences through subsequent aggregation.

### 3.2. Adaptive Sparse Sampling Module

To alleviate the inefficiency caused by uncontrolled neighborhood expansion in large-scale graph computations, we introduce an information flow-aware adaptive sparse sampling module. This module aims to preserve high-influence propagation paths while eliminating structurally redundant or noisy connections. The sampling process is guided by the (IFM) and proceeds in a hierarchical manner.

Given a target node $v_{i}$, the sampling begins with its initial neighborhood $\Gamma^{\left(0\right)}\left(v_{i}\right)=\left\{v_{i}\right\}$, and iteratively constructs higher-order sparse neighborhoods up to a maximum depth K. At each iteration k, the algorithm computes a dynamic threshold:

(3) $$r^{\left(k\right)}=\mu \cdot \mathrm{AVG}\left(a_{j,h}|v_{j}\in \Gamma^{\left(k-1\right)}\left(v_{i}\right),v_{h}\in \Gamma \left(v_{j}\right)\right)$$

where $a_{j,h}$ denotes the propagation intensity from node $v_{j}$ to its neighbor $v_{h}$ based on the IFM, and μ is a sparsification coefficient that controls the selectivity level.

Only neighbors with $a_{j,h}\geq r^{\left(k\right)}$ are retained to form the k-hop sparse set $\Gamma^{\left(k\right)}\left(v_{i}\right)$. To determine whether further expansion is necessary, the algorithm computes the propagation intensity variation:

(4) $$\Delta Q^{\left(k\right)}=\left|Q^{\left(k\right)}-{\bar{Q}}^{\left(k-1\right)}\right|,\;{\bar{Q}}^{\left(k\right)}=\frac{1}{\left|S_{k}\right|}\sum_{\left(v_{j},v_{h}\right)\in S_{k}}a_{j,h}$$

where $S_{k}=\left\{\left(v_{j},v_{h}\right)|v_{j}\in \Gamma^{\left(k\right)}\left(v_{i}\right),v_{h}\in \Gamma \left(v_{j}\right)\right\}$. If $\Delta Q^{\left(k\right)}<\varepsilon$, indicating diminishing new information, the sampling process terminates early.

Finally, the sparse neighborhood is constructed by aggregating all valid layers:

(5) $$\Gamma^{\left(K\right)}\left(v_{i}\right)=\overset{K}{\underset{k=1}{\cup }}\Gamma^{\left(k\right)}\left(v_{i}\right)$$

This dynamic strategy ensures that only structurally meaningful nodes are retained, significantly reducing computational cost and suppressing noisy signals. Empirical results (Appendix A.2, Table A8) confirm that performance is stable across different values of ε. In line with prior findings that structural information is largely contained within low-order neighborhoods [38,39], we set the maximum hop K = 3 and adopt the dataset-specific optimal thresholds reported in Appendix A.2 Table A8 and Table A9 as the default configuration for all experiments.

### 3.3. Hierarchical Multi-Hop Gated Aggregation

To model both local and high-order structural information, we propose a hierarchical multi-hop aggregation framework consisting of two stages: multi-hop attention-based neighborhood fusion and adaptive dual-gating for feature integration.

#### 3.3.1. Decay-Aware Multi-Hop Attention

As defined in Equation (6), we introduce a decay-aware graph attention mechanism to capture the structural dynamics of community structures during information propagation. This mechanism quantifies the topological influence between nodes through an adaptive decay function and leverages the sparsity of attention weights to selectively aggregate neighborhood features that significantly affect a node’s community affiliation.

(6) $$\alpha_{ij}^{\left(k\right)}=\frac{\exp\left(\Phi \left(d_{ij}\right)\cdot \langle W_{q}h_{i},W_{k}h_{j}\rangle \right)}{\sum_{l\in \Gamma^{\left(k\right)}\left(v_{i}\right)}\exp\left(\Phi \left(d_{il}\right)\cdot \langle W_{q}h_{i},W_{k}h_{l}\rangle \right)}$$

Here, $h_{i}$ and $h_{j}$ denote the feature vectors of nodes i and j, respectively; $\Gamma^{\left(k\right)}(v_{i})$ represents the k-hop neighborhood of node i; and $d_{ij}$ is the shortest-path distance between nodes i and j. The decay function $\Phi (d_{ij})=e^{-\lambda d_{ij}}$ emphasizes contributions from short-path neighbors while reducing interference from distant nodes. $W_{q}$ and $W_{k}$ are learnable projection matrices used to model the structural similarity between node features.

To mitigate the gradient vanishing problem, as shown in Equation (7), we apply layer normalization to smooth and calibrate aggregated feature distributions, thereby improving training stability.

(7) $$h_{i}^{\left(k\right)}=\;\mathrm{LayerNorm}\;\left(\sum_{j\in \Gamma^{\left(k\right)}\left(v_{i}\right)}\alpha_{ij}^{\left(k\right)}W_{v}h_{j}\right)$$

#### 3.3.2. Adaptive Feature Fusion via Dual-Gating

To address the imbalance between local topological features and global path information caused by static weight allocation in traditional community detection methods, we introduce a two-level gating mechanism guided by prior knowledge and data distribution feedback. This mechanism dynamically adjusts the contribution of local and high-order features to enable adaptive decision-making for multi-order representations.

(1) Prior Knowledge-Guided Initial Gating:

We generate the initial gating weights using node degree and local information flow intensity to quantify each node’s structural importance within its local community, as defined in Equation (8). This ensures that local topological features are effectively captured.

(8) $$g^{\left(\mathrm{base}\;\right)}=\mathrm{softmax}\left(W_{r}\cdot \left[\deg\left(v_{i}\right),\max\left(m_{i}\right)\right]\right)$$

Here, $\deg\left(v_{i}\right)$ denotes the degree of node $v_{i}$, quantifying its connection density in the local community, and $\max\left(m_{i}\right)$ represents the maximum information flow intensity in the neighborhood of $v_{i}$. The learnable routing matrix $W_{r}\in {\mathbb{R}}^{K\times 2}$ maps scalar priors to the K-hop feature weight space.

(2) Data Distribution Feedback-Based Gating:

To adaptively refine the gating weights, we incorporate batch-wise feature statistics, as shown in Equation (9). This feedback mechanism adjusts the gating based on empirical feature distribution, improving feature representation and model generalization.

(9) $$\Delta g=\tanh\left(W_{f}\cdot \mathrm{Std}\left(H_{\mathrm{batch}}\right)\right),\;g^{\left(\mathrm{final}\;\right)}=g^{\left(\mathrm{base}\;\right)}⊙\Delta g$$

Here, $\mathrm{Std}\left(H_{\mathrm{batch}\;}\right)\in {\mathbb{R}}^{d}$ denotes the standard deviation vector of node features in the current batch, quantifying the distribution dispersion across feature dimensions. The feedback projection matrix $W_{f}\in {\mathbb{R}}^{K\times d}$ compresses high-dimensional statistics into the gating weight space, and ⊙ represents the Hadamard product.

We further apply a sparsification mapping, as defined in Equation (10), to suppress redundant signals and promote generalization.

(10) $$y_{i}=\mathrm{MLP}\left(\sum_{k=1}^{K}g_{k}^{\left(\mathrm{final}\;\right)}\cdot h_{i}^{\left(k\right)}\right)$$

During model training, the cross-entropy loss for community partitioning and the sparsity regularization term of the gating mechanism are jointly optimized to enhance the model’s expressive power and generalization performance. The overall loss function is defined as shown in Equation (11):

(11) $$L=L_{\mathrm{CE}}+\kappa \sum_{k=1}^{K}{\left\|g_{i,k}^{\mathrm{final}}\right\|}_{1}$$

Here, $L_{\mathrm{CE}}$ denotes the cross-entropy loss, which measures the discrepancy between model predictions and ground-truth labels; κ is the regularization coefficient that controls the strength of the sparsity regularization term; and $g_{i,k}^{\mathrm{final}\;}$ denotes the gating weight of the *k*-th hop for node *i*, used to adaptively control the contribution of each hop-level feature. The associated sparsity regularization encourages the model to retain only essential hops, enhancing interpretability and robustness.

## 4. Experiments and Results

### 4.1. Datasets

We conduct experiments using seven real-world network datasets and two synthetic networks generated by the LFR benchmark model. Detailed descriptions of these datasets are summarized in Table 1.

### 4.2. Evaluation Metrics

We evaluate the performance of the DAMA model in community detection using the following metrics, summarized in Table 2.

Here, N denotes the total number of nodes; $y_{i}$ and ${\hat{y}}_{i}$ represent the true and predicted labels of node i, respectively; I is an indicator function; $I(y;\hat{y})$ represents mutual information; H(y) and $H(\hat{y})$ are the entropies of the true and predicted labels, respectively. RI is the Rand Index, and E[RI] is its expected value under random labeling; $A_{ij}$ is the adjacency matrix of the graph; $k_{i}$ and $k_{j}$ are the degrees of nodes i and j. m is the total number of edges; and $\delta \left(c_{i},c_{j}\right)$ is an indicator function equal to 1 if nodes i and j belong to the same community and 0 otherwise. In our experimental setup, each experiment is repeated 50 times, and the average values of the above metrics are reported to ensure the stability and reliability of the results.

### 4.3. Experimental Setup

The parameter configuration of the proposed DAMA model comprises a multi-hop graph attention layer, a gating mechanism module, and a classifier module. Specifically, the multi-hop graph attention layer employs 8 attention heads, each outputting 16-dimensional features, which are aggregated to form a 128-dimensional node representation. During message passing, a learnable decay coefficient λ is introduced to modulate the influence of neighborhoods based on shortest-path distances.

The gating mechanism module integrates 8 attention heads to achieve adaptive fusion based on node degree distribution and batch-wise statistical features, followed by a two-layer MLP (128 → 128) with ReLU activation. The classifier module consists of a two-layer MLP (128 → 64 → number of classes) also utilizing ReLU activation. All graph attention layers adopt ELU activation functions, and a dropout rate of 0.6 is maintained throughout the training process.

The model is trained using the Adam optimizer with a learning rate of 0.001 and weight decay of 1 × 10^−4^. The training process is fixed for 200 epochs.

For the supervised community detection task, we adopt a standard transductive learning setup: nodes are randomly split into 80% for training and 20% for testing, with binary masks ensuring that the loss is computed only on the training set and all reported metrics are evaluated exclusively on the test set. This guarantees strict separation between the training and evaluation processes. This partitioning scheme is validated through 50 independent repeated experiments with different random seeds, and the average results across all experiments are reported to ensure statistical reliability and prevent information leakage between the training and evaluation phases.

### 4.4. Hyperparameter Experiments

We evaluate the impact of the sparsification strength parameter μ and the information decay parameter λ on the model’s performance and stability on the Cora dataset using a grid search strategy. Both μ and λ are sampled from the range [0.1, 1.0] with a step size of 0.1. Each combination is tested over 20 independent runs.

The optimal hyperparameter settings may vary across datasets. However, due to the substantial computational cost of exhaustive hyperparameter tuning on all datasets, we performed this process only on the representative Cora dataset. The configuration identified on Cora was then directly applied to all other datasets. The consistently superior results achieved by our model (as shown in the comparative experiments of Figure 6) confirm the strong generalizability and robustness of this hyperparameter set across diverse data.

The mean values and standard deviations of the Accuracy, NMI, and F1 metrics were calculated. The experimental results are illustrated in Figure 3.

The mean values and standard deviations of Accuracy, NMI, F1, ARI, and Modularity were calculated, and the experimental results are shown in Figure 3. It can be observed that the model performs well when μ is in the ranges [0.2–0.3, 0.8–1.0], and all metrics tend to improve as μ increases. However, the current experimental results are limited by the upper bound of μ=1.0, which restricts exploration of the parameter space’s global characteristics.

**Figure 4:**
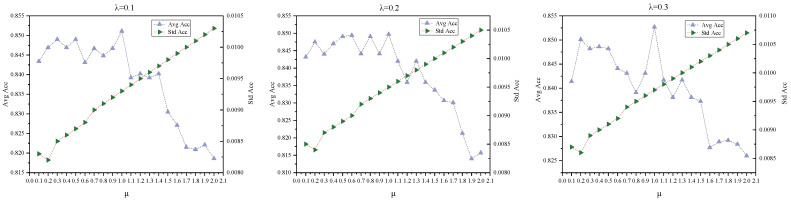
Analysis of Model Behavior under Extended Sparsification Strength Parameter Ranges.

To address this limitation, we extended the sparsification parameter range in subsequent experiments. Specifically, we fixed λ∈[0.1,0.3] (a low-variance interval, relatively stable) and increased the upper bound of μ to 2 to investigate the model’s behavior under extreme sparsification, using the ACC metric for evaluation (as it is representative and provides an intuitive reflection of overall performance). These results are shown in Figure 4.

The following conclusions can be drawn from Figure 3 and Figure 4:

(1) As shown in the upper layer of Figure 3 and Figure 4, the model exhibits notable performance fluctuations when μ<1, but the best results are consistently achieved at μ=1; further increasing μ leads to a performance decline. Within the range μ∈[0.1,1.0], the combined effect of μ and λ keeps fluctuations in Accuracy, F1-score, and ARI within approximately ±2%. However, due to its sensitivity to class alignment, NMI exhibits larger fluctuations of up to ±4%. Modularity reaches its maximum when μ=1.
(2) As shown in the lower layer of Figure 3 and Figure 4, the sparsification parameter μ affects both model performance and stability. Overall, the standard deviations of the evaluation metrics are small: Accuracy, NMI, F1-score, and Modularity all remain below 0.004, while ARI exhibits a higher standard deviation of approximately 0.02. As μ increases, the standard deviations of Accuracy, NMI, and F1-score show a slight upward trend. This effect is likely due to the increased sparsity induced by the adaptive sparse sampling module at higher μ, which amplifies divergence in input feature distributions and consequently increases output uncertainty. ARI and Modularity, in contrast, show irregular fluctuations because they depend on exact label matching and the global network structure. Overall, increasing μ can improve certain performance metrics but may reduce stability. Therefore, for applications that require stable outputs, it is advisable to select relatively lower intervals of μ.

**Figure 5:**
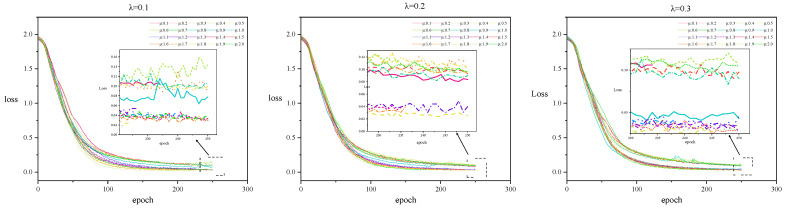
Convergence Trends of the Loss Function under Different Sparsification Strengths.

To validate the convergence characteristics of the model in the extended parameter space, we retain the configuration λ∈[0.1,0.3] from Figure 4 and record the epoch-wise evolution of the training loss under different μ values. The results are presented in Figure 5.

The following conclusions can be drawn from Figure 5:

(1) During the initial training phase (up to 200 epochs), the loss curves for different μ values largely overlap, suggesting that the sparsification strength does not significantly affect the convergence trajectory in the early stages. However, after 200 epochs, the loss curves begin to exhibit non-monotonic fluctuations, indicating potential overfitting or instability.
(2) Comparing different sparsification settings shows that when μ<1, the final loss values remain consistently higher than those observed in the μ>1 setting. We hypothesize that larger μ values simplify the data representation by increasing the intensity of feature filtering, which in turn facilitates faster convergence and easier optimization.
(3) The training process follows a typical three-phase pattern: in the rapid convergence phase (epochs < 100), loss decreases sharply; in the fine optimization phase (100 ≤ epochs ≤ 200), the rate of loss reduction slows; and in the overfitting risk phase (epochs > 200), loss fluctuates unpredictably. We recommend training for 180–200 epochs to balance convergence and generalization.

### 4.5. Comparative Experiments

**Figure 6:**
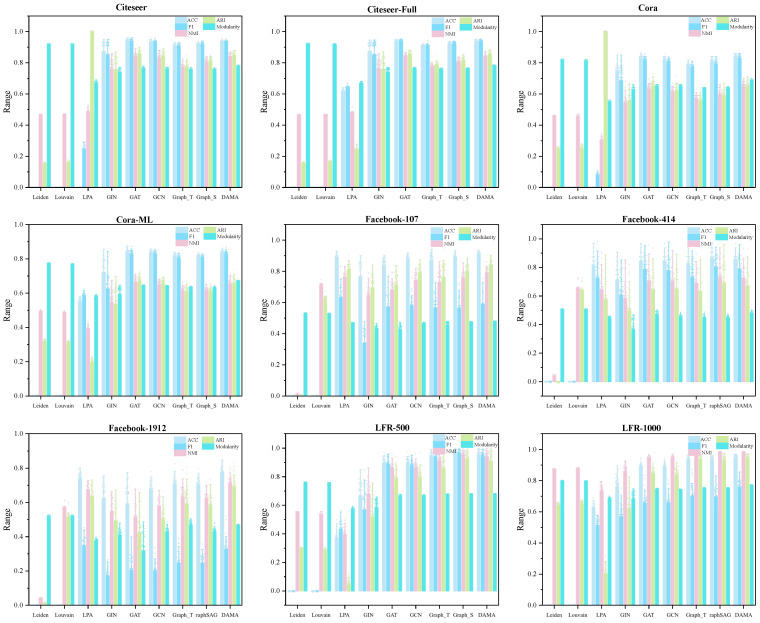
Performance comparison of DAMA and baseline models across datasets. Error bars indicate the standard deviation over 50 runs, with shorter bars reflecting higher stability.

To comprehensively evaluate the effectiveness of the proposed DAMA model across diverse graph learning scenarios, we conduct comparative experiments against multiple baseline models, covering both GNN-based and traditional community detection approaches (Table 3). The baselines include models employing local aggregation, global reasoning, inductive learning, label propagation, and modularity optimization. To ensure fair comparison, all GNN baselines (GCN, GAT, GraphSAGE, GIN) are implemented with 2 layers, 64 hidden units, and trained using the same optimizer settings (Adam with learning rate 0.001) and data splitting strategy as DAMA. For traditional methods (Louvain, Leiden, LPA), we use the implementations provided by the original authors with default parameters as recommended in their respective publications. The comparative experimental results are shown in Figure 6. In the figure, Graph_S represents GraphSAGE, and Graph_T represents Graph Transformer.

The following conclusions are drawn based on Figure 6 and the results summarized in Table 1:

In terms of overall performance, the proposed DAMA method demonstrates significant advantages across multiple evaluation metrics. Experimental results indicate that DAMA achieves the best or near-best comprehensive performance across different types of datasets. Notably, DAMA consistently attains higher modularity scores compared to other graph neural network methods, highlighting its superior capability in capturing community structures. Moreover, the model exhibits relatively stable performance across multiple runs, reflecting its robust behavior.

When compared with baseline GNN models, clear performance differences are observed. GAT and GCN achieve comparable and stable results across most datasets, but their overall performance remains slightly lower than DAMA. GIN shows considerable fluctuations on small-sample or high-noise datasets, underscoring its limitations in capturing multi-hop dependencies. GraphSAGE and GraphTransform perform well on large-scale dense graphs, yet slightly underperform compared to GAT and GCN on small-scale or sparse graphs, indicating that different GNN architectures have distinct advantages depending on graph characteristics.

Traditional community detection methods exhibit limited performance in this comparison. Leiden and Louvain algorithms, due to over-optimization of modularity, perform poorly on external evaluation metrics and do not involve node label prediction, thus failing to provide classification metrics such as ACC and F1. The label propagation algorithm (LPA) performs poorly on sparse or structurally complex graphs and shows limited effectiveness.

From the perspective of dataset characteristics and model adaptability, in sparse graphs, GAT and GCN effectively leverage local neighborhood information, while DAMA further improves performance. LPA and other traditional methods exhibit comparatively weaker performance in such graphs. On large-scale community-structured graphs, GraphSAGE, GraphTransform, and DAMA achieve prominent results in ACC, NMI, and ARI, whereas the instability of GIN reflects its limited capacity in capturing long-range dependencies. In social network graphs, where node degree distributions are highly uneven, all models face greater challenges. Nevertheless, DAMA maintains stable performance across all metrics and achieves notably higher modularity than other methods, further confirming its superiority in capturing community structures.

### 4.6. Ablation Experimental

To evaluate the effectiveness of the core components in the DAMA framework, we conduct comprehensive ablation studies on the Information Flow Matrix (IFM), the Adaptive Sparse Sampling (ASS), and the Hierarchical Multi-Hop Gated Aggregation (MSG). By examining performance variations after removing each individual component, we assess their adaptability to sparse, dense, and modular networks, and further reveal the intrinsic relationships between graph topological characteristics and architectural design. The detailed experimental results are systematically reported in Table 4.

Based on the dataset characteristics in Table 1 and the ablation results in Table 4, we derive the following observations. For all tables, the boldfaced values represent the relatively superior results:

The core modules of the DAMA model exhibit complementary roles across different graph structures. The IFM is primarily responsible for constructing global node representations and enhancing structural discrimination. Ablation results show that removing IFM leads to the most pronounced performance degradation across most datasets (e.g., CiteSeer, Cora, Cora-ML, Facebook 414, LFR-1000), highlighting its key role in integrating global features.

The ASS proves particularly effective in dense and modular graphs, where it dynamically adjusts the receptive field to filter redundant connections and reinforce community boundaries. Its impact is clearly dataset-dependent: in graphs such as CiteSeer and Cora-ML, removing ASS results in noticeable performance drops, indicating its importance for structure optimization and denoising in dense or modular networks. In contrast, in graphs like Facebook 107 and Facebook 1912, its removal leads to minor changes or slight fluctuations, suggesting that its contribution depends on the intrinsic structural characteristics of the graph.

The MSG enhances model robustness by integrating multi-scale feature information. Across datasets including CiteSeer, Cora, and LFR-1000, removing MSG causes visible performance decreases, with the effect on CiteSeer comparable to that of IFM. This indicates that MSG plays a key role in capturing graph structural information at multiple scales, and its absence can limit the model’s comprehensive understanding of the graph, thus affecting overall representation and generalization.

In summary, IFM, ASS, and MSG function complementarily: IFM supports global information integration, MSG strengthens cross-scale feature capture, and ASS optimizes local structure awareness and sampling strategies. This complementary design ensures that DAMA can dynamically adapt to diverse graph topologies, maintaining robust performance and high generalization across sparse, dense, and modular networks.

### 4.7. Noise Robustness Experiments

To thoroughly evaluate the robustness of our model under noisy environments, we introduce various perturbation mechanisms into the Cora dataset to simulate realistic graph noise scenarios. Special emphasis is placed on assessing the effectiveness of the adaptive sparse sampling module in suppressing structural disturbances while preserving essential topological information.

Noise is injected from two perspectives—structural noise and feature (label) noise—with perturbation ratios ranging from 0.1 to 0.5. This indicates that 10% to 50% of the total edges or labels are subject to modification. The types of noise and their corresponding design purposes are summarized in Table 5.

**Figure 7:**
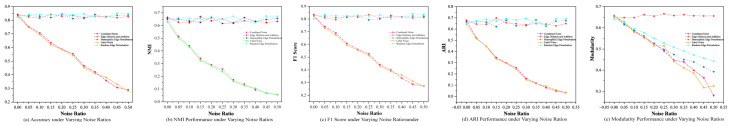
Model performance under different types and intensities of noise.

Under the above settings, experiments are conducted for each type of noise at various perturbation intensities. The results are illustrated in Figure 7.

Based on the results in Figure 7, the following observations can be made:

(1) As the intensity of structural noise increases, the model exhibits only a marginal decline in both Accuracy and F1-score, indicating strong robustness against structural perturbations. Meanwhile, although the Adjusted Rand Index (ARI) shows relatively larger fluctuations, it generally stabilizes at a consistent level. Modularity generally decreases with increasing noise, except in the Edge Deletion and Addition scenario, where changes are relatively small as the community structure remains largely intact. This robustness can be primarily attributed to the adaptive sparse sampling module, which effectively identifies and filters out redundant or anomalous edges, thereby significantly mitigating the adverse impact of structural noise and preserving the essential topological properties of the graph.
(2) In contrast, under label noise and combined noise scenarios, the model experiences a substantial degradation in Accuracy, Normalized Mutual Information (NMI), F1-score, and ARI as the noise intensity increases. This observation suggests that the model has limited tolerance to label corruption and lacks a robust correction mechanism for noisy annotations. These findings underscore the critical importance of high-quality labels in constructing accurate classification boundaries. Furthermore, under combined noise settings, the performance deterioration closely mirrors that observed under label noise alone, indicating that label corruption serves as the dominant factor driving model performance degradation in complex noise environments.

**Figure 8:**
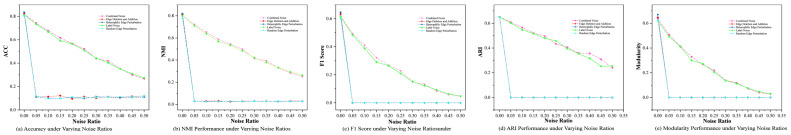
Robustness comparison before and after removing the adaptive sparse sampling module.

To further verify the role of the adaptive sparse sampling module, a comparative experiment was conducted in which this module was removed while all other configurations remained unchanged. The performance in terms of Accuracy, NMI, F1-score, ARI, and Modularity under varying noise intensities is shown in Figure 8.

A comparison between Figure 7 and Figure 8 reveals that, after removing the adaptive sparse sampling module, the model’s performance degrades significantly under structural perturbations. These results provide strong evidence that the adaptive sparse sampling mechanism plays a vital role in alleviating structural information corruption and improving the model’s robustness.

## 5. Conclusions

To bridge the gap between structural topology and information flow in static graphs, we propose DAMA, a model that captures multi-scale, dynamic propagation patterns to enhance structural modeling and suppress noisy or irrelevant neighbors.

Extensive experiments on both real-world and synthetic graphs demonstrate that DAMA consistently outperforms representative baseline models across multiple evaluation metrics, including Accuracy, NMI, F1-score, ARI, and Modularity. Moreover, the model’s explicit modeling of propagation strength and adaptive sampling contributes to its stronger robustness and better interpretability. Ablation and sensitivity analyses further confirm the effectiveness of each component and the reliability of the gating mechanism. In future work, we plan to extend DAMA to dynamic or attributed graphs and explore its application in unsupervised and online community detection scenarios.

## Figures and Tables

**Table 1:** Summary of the benchmark datasets used in this study.

Dataset Name	Nodes	Edges	Avg Degree	Diameter	Avg Path Length	Avg Clustering Coefficient	Modularity
CiteSeer [40]	3327	4676	2.81	28	9.32	0.257	0.886
CiteSeer-full [40]	4230	5337	2.52	26	7.36	0.243	0.918
Cora [41]	2708	5278	3.89	19	6.31	0.293	0.809
Cora-ML [41]	2995	8158	5.44	17	5.27	0.342	0.761
Facebook 107 [42]	1047	27,755	53.12	2	1.94	0.852	0.526
Facebook 414 [42]	159	3670	23.08	2	1.85	0.686	0.507
Facebook1912 [42]	755	30,742	81.43	2	1.89	0.669	0.522
LFR-500 [43]	500	1498	5.99	13	5.35	0.534	0.742
LFR-1000 [43]	1000	7185	14.37	7	4.05	0.601	0.787

**Table 2:** Evaluation Metrics in This Paper.

Evaluation Metric	Definition	Calculation Formula
Accuracy [44]	Measures the proportion of correctly classified node labels.	$\mathrm{Accuracy}\;=\frac{1}{N}\sum_{i=1}^{N}I\left(y_{i}={\hat{y}}_{i}\right)$
Normalized Mutual Information (NMI) [44]	Quantifies the alignment between predicted and ground-truth community partitions.	$\mathrm{NMI}=\frac{2\cdot I(y;\hat{y})}{H(\hat{y})+H(\hat{y})}$
F1 Score	The harmonic mean of precision and recall, balancing sensitivity and specificity.	$\mathrm{Fl}=2\cdot \frac{\;\mathrm{Precision}\;\cdot \;\mathrm{Recall}\;}{\;\mathrm{Precision}\;+\;\mathrm{Recall}\;}$
Adjusted Rand Index (ARI) [45]	Measures agreement between predicted and true labels	$\mathrm{ARI}=\frac{\mathrm{RI}-E[\mathrm{RI}]}{\max(\mathrm{RI})-E[\mathrm{RI}]}$
Modularity [7]	Evaluates density of edges within predicted communities.	$Q=\frac{1}{2m}\sum_{i,j}\left[A_{ij}-\frac{k_{i}k_{j}}{2m}\right]\delta \left(c_{i},c_{j}\right)$

**Table 3:** Summary of core mechanisms and technical paradigms of compared models.

Model	Core Mechanism	Technical Paradigm
Louvain [20]	Modularity optimization	Modularity maximization
Leiden [10]	Modularity optimization with refinement	Modularity maximization and structural refinement
LPA [46]	Label propagation among neighbors	Label propagation
GCN [47]	Spectral graph convolution	Local feature aggregation
GAT [48]	Attention-based neighbor aggregation	Local aggregation via attention mechanism
GraphSAGE [32]	Inductive neighborhood sampling	Inductive learning with localized aggregation
GIN [49]	MLP-based aggregation with structural encoding	Structure-aware graph representation
GraphTransformer [50]	Positional encoding with global attention	Global relational modeling

**Table 4:** Ablation Study Results.

**Model**	**Citeseer**					**Citeseer_Full**					**Cora**				
	**ACC**	**NMI**	**F1**	**ARI**	**Mod**	**ACC**	**NMI**	**F1**	**ARI**	**Mod**	**ACC**	**NMI**	**F1**	**ARI**	**Mod**
DAMA–IFM	0.611	0.341	0.509	0.310	0.550	0.844	0.700	0.851	0.649	0.751	0.807	0.600	0.792	0.608	0.641
DAMA–ASS	0.69	0.418	0.568	0.435	0.616	0.933	0.832	0.935	0.843	0.771	0.828	0.635	0.813	0.651	0.670
DAMA–MSG	0.717	0.441	0.580	0.459	0.603	0.933	0.832	0.934	0.842	0.736	0.845	0.663	0.833	0.682	0.617
DAMA	**0.725**	**0.464**	**0.586**	**0.477**	**0.708**	**0.936**	**0.838**	**0.938**	**0.850**	**0.788**	**0.849**	**0.669**	**0.836**	**0.687**	**0.689**
**Model**	**Cora-ML**					**Facebook 107**					**Facebook 414**				
	**ACC**	**NMI**	**F1**	**ARI**	**Mod**	**ACC**	**NMI**	**F1**	**ARI**	**Mod**	**ACC**	**NMI**	**F1**	**ARI**	**Mod**
DAMA–IFM	0.826	0.637	0.818	0.640	0.637	**0.910**	**0.799**	**0.595**	**0.848**	**0.478**	0.867	0.823	0.804	0.728	0.520
DAMA–ASS	0.842	0.663	0.831	0.673	0.663	0.904	0.789	0.575	0.832	0.476	0.857	0.824	0.801	0.717	0.505
DAMA–MSG	0.844	0.663	0.835	0.671	**0.677**	0.907	0.793	0.592	0.845	0.476	0.863	0.815	0.800	0.719	0.518
DAMA	**0.850**	**0.674**	**0.840**	**0.688**	0.565	0.904	0.789	0.575	0.832	0.476	**0.873**	**0.829**	**0.809**	**0.741**	**0.522**
**Model**	**Facebook 1912**					**LFR-500**					**LFR_1000**				
	**ACC**	**NMI**	**F1**	**ARI**	**Mod**	**ACC**	**NMI**	**F1**	**ARI**	**Mod**	**ACC**	**NMI**	**F1**	**ARI**	**Mod**
DAMA–IFM	0.770	0.710	0.323	0.686	0.458	0.946	0.930	0.938	0.892	0.675	0.937	0.969	0.749	0.917	0.768
DAMA–ASS	**0.778**	**0.716**	0.316	**0.706**	0.455	**0.957**	**0.944**	**0.950**	**0.914**	0.675	0.936	0.976	0.700	0.933	0.754
DAMA–MSG	0.769	0.710	0.321	0.686	0.458	0.947	0.932	0.938	0.894	0.675	0.940	0.971	0.751	0.921	0.768
DAMA	0.773	0.713	**0.326**	0.692	**0.462**	0.952	0.940	0.942	0.908	**0.678**	**0.948**	**0.978**	**0.757**	**0.940**	**0.770**

**Table 5:** Classification and description of network noise types.

Noise Category	Perturbation Mode	Description
Structural Noise	Random Edge Perturbation	Randomly adds unrelated edges to simulate erroneous or redundant connections.
Structural Noise	Heterophilic Edge Perturbation	Adds edges between nodes of different labels to disturb community boundaries.
Structural Noise	Edge Deletion and Addition	Deletes part of the original edges and injects label-differing edges to simulate compound structural interference.
Feature Noise	Label Noise	Randomly corrupts node labels to simulate annotation errors or noise.
Combined Noise	Combined Noise	Applies both structural and label noise simultaneously to simulate real-world complex perturbation scenarios.

## Data Availability

The datasets used in this study include publicly available benchmark datasets (e.g., CiteSeer, Cora, and Facebook networks), which are cited and referenced in the manuscript. The synthetic LFR benchmark datasets were generated using publicly available code, and the generation scripts can be provided upon reasonable request from the corresponding author. The original datasets are available from the following sources: CiteSeer/Cora/Cora-ML—https://linqs.soe.ucsc.edu/data, Facebook datasets (ego-networks)—https://snap.stanford.edu/data/egonets-Facebook.html, and LFR benchmark graphs—https://github.com/eXascaleInfolab/LFR-Benchmark_UndirWeightOvp (accessed on 12 March 2025).

## Appendix A

### Appendix A.1. Ablation Study on β and γ

To evaluate the sensitivity of our model to the hyperparameters β and γ, we performed a grid search over the range of 0.1 to 1.0 on the Cora dataset. The top 10% performing combinations for each metric are highlighted in bold and presented in Table A1, Table A2, Table A3, Table A4 and Table A5, while Table A6 summarizes the frequency of these combinations. Considering the overall performance across metrics and the most frequently occurring combinations, we select (β=0.4,γ=0.7) as the optimal configuration for the Cora dataset. The optimal combinations for all datasets are listed in Table A7.

**Table A1:** Accuracy for varying values of β and γ on the Cora dataset.

β\γ	0.10	0.20	0.30	0.40	0.50	0.60	0.70	0.80	0.90	1.00
0.10	0.8158	0.8402	0.8417	0.8468	0.8417	0.8472	0.8480	0.8505	0.8321	0.8480
0.20	0.8251	0.8472	0.8524	0.8398	0.8576	0.8454	0.8428	0.8557	0.8443	0.8472
0.30	0.8350	0.8517	0.8531	0.8468	0.8406	0.8535	0.8487	0.8520	0.8480	**0.8579**
0.40	0.8362	0.8579	0.8572	0.8424	0.8417	0.8487	**0.8682**	0.8498	0.8612	0.8480
0.50	0.8258	0.8583	0.8553	0.8476	0.8450	0.8576	0.8561	0.8528	0.8565	0.8509
0.60	0.8421	0.8550	0.8605	0.8447	0.8439	**0.8631**	0.8502	0.8505	0.8468	0.8572
0.70	0.8354	0.8535	0.8561	0.8539	**0.8664**	0.8469	**0.8664**	0.8413	0.8487	0.8576
0.80	0.8255	0.8339	**0.8576**	0.8436	0.8557	0.8491	0.8395	0.8402	0.8520	0.8495
0.90	0.8277	0.8376	0.8432	**0.8598**	0.8461	0.8565	0.8557	**0.8627**	0.8579	0.8369
1.00	0.8288	0.8443	0.8472	0.8505	0.8531	0.8561	0.8531	**0.8616**	**0.8594**	0.8494

**Table A2:** NMI for varying values of β and γ on the Cora dataset.

β\γ	0.10	0.20	0.30	0.40	0.50	0.60	0.70	0.80	0.90	1.00
0.10	0.6298	0.6532	0.6531	0.6660	0.6590	0.6664	0.6639	0.6746	0.6429	0.6677
0.20	0.6358	0.6737	0.6802	0.6512	0.6798	0.6580	0.6613	**0.6870**	0.6612	0.6651
0.30	0.6545	0.6722	0.6818	0.6674	0.6597	0.6802	0.6686	0.6706	0.6624	0.6840
0.40	0.6523	**0.6879**	0.6813	0.6548	0.6552	0.6648	**0.7007**	0.6698	**0.6936**	0.6640
0.50	0.6358	0.6844	0.6810	0.6670	0.6663	**0.6877**	0.6824	0.6716	0.6832	0.6711
0.60	0.6576	0.6837	0.6912	0.6631	0.6605	**0.6981**	0.6711	0.6712	0.6632	0.6851
0.70	0.6545	0.6771	0.6824	0.6823	**0.7024**	0.6623	**0.7050**	0.6514	0.6655	0.6819
0.80	0.6326	0.6474	0.6882	0.6569	0.6811	0.6680	0.6578	0.6546	0.6768	0.6675
0.90	0.6398	0.6517	0.6562	**0.6915**	0.6599	0.6795	0.6798	0.6860	**0.6887**	0.6464
1.00	0.6335	0.6640	0.6651	0.6716	0.6777	0.6841	0.6756	**0.6908**	**0.6900**	0.6664

**Table A3:** F1-score for varying values of β and γ on the Cora dataset.

β\γ	0.10	0.20	0.30	0.40	0.50	0.60	0.70	0.80	0.90	1.00
0.10	0.7878	0.8232	0.8244	0.8282	0.8295	0.8348	0.8308	**0.8387**	0.8224	0.8383
0.20	0.8074	0.8352	0.8396	0.8280	**0.8459**	0.8324	0.8270	**0.8507**	0.8361	0.8381
0.30	0.8163	0.8397	0.8424	0.8369	0.8380	0.8411	0.8390	0.8474	0.8374	0.8497
0.40	0.8210	0.8480	0.8438	0.8318	0.8318	0.8371	**0.8622**	0.8380	0.8547	0.8398
0.50	0.8140	0.8421	0.8433	0.8377	0.8348	0.8469	0.8423	0.8424	0.8471	0.8393
0.60	0.8287	0.8428	**0.8534**	0.8332	0.8333	**0.8534**	0.8378	0.8395	0.8384	0.8459
0.70	0.8288	0.8442	0.8474	0.8453	**0.8573**	0.8390	**0.8590**	0.8273	0.8384	0.8483
0.80	0.8193	0.8192	0.8451	0.8291	0.8494	0.8416	0.8293	0.8295	0.8431	0.8391
0.90	0.8150	0.8271	0.8295	0.8450	0.8371	0.8419	0.8481	**0.8510**	**0.8513**	0.8272
1.00	0.8182	0.8319	0.8326	0.8388	0.8456	**0.8525**	0.8460	0.8500	**0.8534**	0.8351

**Table A4:** ARI for varying values of β and γ on the Cora dataset.

β\γ	0.10	0.20	0.30	0.40	0.50	0.60	0.70	0.80	0.90	1.00
0.10	0.4732	0.6104	0.6348	0.6813	0.6529	0.6841	**0.7040**	**0.7186**	0.6745	0.664
0.20	0.5572	0.5848	0.618	0.6488	0.6806	0.6715	**0.6969**	0.6951	0.6745	0.6659
0.30	0.5476	0.608	0.6137	0.6534	0.6271	0.6801	0.6619	0.6759	0.6717	0.6738
0.40	0.5805	0.6142	0.6636	0.6727	0.6621	0.6826	**0.6946**	0.6903	**0.7041**	0.6689
0.50	0.5958	0.6038	0.6466	0.6667	0.6704	0.6622	0.6848	0.6648	0.6626	0.6751
0.60	0.6154	0.6421	0.6543	0.6270	0.6694	**0.6982**	0.6842	0.6842	0.6700	0.6703
0.70	0.6246	0.6241	0.6332	0.6708	0.6417	0.6683	0.6650	0.6708	0.6587	**0.6896**
0.80	0.6154	0.6524	0.6439	0.6156	0.6645	0.6788	0.6800	0.6822	0.6798	0.6516
0.90	0.6533	0.5842	0.6285	0.6395	0.6467	**0.7007**	0.6503	0.6809	0.6683	**0.6904**
1.00	0.6158	0.6113	0.6117	0.6504	0.6497	0.6383	0.6842	0.6609	**0.6967**	0.6855

**Table A5:** Modularity for varying values of β and γ on the Cora dataset.

β\γ	0.10	0.20	0.30	0.40	0.50	0.60	0.70	0.80	0.90	1.00
0.10	0.6511	0.6788	0.6836	0.6917	**0.6932**	0.6917	0.6898	0.6898	0.6877	0.6879
0.20	0.6722	0.6879	0.6915	0.6899	**0.6960**	**0.6923**	0.6910	0.6903	0.6877	0.6905
0.30	0.6791	0.6855	0.6898	0.6932	0.6917	**0.6939**	0.6907	0.6892	0.6919	0.6849
0.40	0.6846	0.6894	0.6924	**0.6943**	**0.6934**	0.6907	**0.6936**	0.6875	0.6874	0.6875
0.50	**0.6886**	0.6852	0.6920	0.6905	**0.6944**	**0.6935**	0.6919	0.6903	0.6895	0.6875
0.60	0.6881	0.6893	0.687	0.6905	0.6905	0.6913	0.6912	0.6889	0.6862	0.6908
0.70	0.6886	0.6866	**0.6929**	0.6882	0.6879	0.6919	0.6908	0.6905	0.6877	0.6888
0.80	0.6859	0.6894	0.6895	0.6895	0.6908	0.6895	0.6906	0.6875	0.6907	0.6882
0.90	0.6865	0.684	0.6893	0.6902	0.6887	0.6908	0.6852	0.6909	0.6887	0.6884
1.00	0.6863	0.6885	0.6878	0.6894	0.6883	0.6896	0.6898	0.6887	0.6886	0.6895

**Table A6:** Best Performing Parameter Combinations on the Cora dataset.

Combination (β, γ)	Times	Accuracy	NMI	F1 Score	ARI	Modularity
**(0.40, 0.70)**	5	0.8682	0.7007	0.8622	0.6946	0.6936
(0.90, 0.10)	4	0.8594	0.6900	0.8534	0.6967	0.6886
(0.60, 0.60)	4	0.8631	0.6981	0.8534	0.6982	0.6913
(0.70, 0.70)	3	0.8664	0.705	0.859	0.665	0.6908
(0.70, 0.50)	3	0.8664	0.7024	0.8573	0.6417	0.6879

**Table A7:** Optimal (β,γ) Combinations for Each Dataset.

Dataset Name	Optimal Parameter Combination (β, γ)
CiteSeer	(0.1, 1.0)
CiteSeer-full	(0.1, 0.7)
Cora	(0.4, 0.7)
Cora-ML	(0.4, 0.9)
Facebook 107	(0.2, 0.8)
Facebook 414	(0.3, 0.8)
Facebook1912	(0.5, 0.6)
LFR-500	(0.2, 1.0)
LFR-1000	(0.4, 0.7)

### Appendix A.2. Sensitivity Analysis of the Sampling Termination Threshold ε

To evaluate the impact of the sampling termination threshold ε on model performance, we conducted experiments over the range {0.01,0.02,…,0.10}. Results on the Cora dataset are summarized in Table A8, indicating that all metrics (Accuracy, NMI, F1 Score, ARI, and Modularity) exhibit stable performance across different ε values. While ε=0.05 achieves the highest Accuracy and NMI on Cora, the optimal threshold varies across datasets, as detailed in Table A9. These empirical findings guide the selection of dataset-specific ε values in subsequent experiments.

**Table A8:** Model performance metrics on the Cora dataset under different values of the sampling termination threshold.

ε	Accuracy	NMI	F1 Score	ARI	Mod
0.01	0.8462	0.6646	0.8341	0.6888	0.7272
0.02	0.8479	0.6677	0.8356	0.6922	0.7278
0.03	0.8479	0.6676	0.8355	0.6928	0.7271
0.04	0.8478	0.6675	0.8354	0.6929	0.7271
**0.05**	**0.8481**	**0.6680**	**0.8357**	0.6969	0.7276
0.06	0.8478	0.6675	0.8354	0.6938	0.7266
0.07	0.8480	0.6678	0.8356	**0.6979**	**0.7283**
0.08	0.8479	0.6676	0.8355	0.6874	0.7276
0.09	0.8478	0.6675	0.8354	0.6939	0.7267
0.10	0.8478	0.6676	0.8354	0.6959	0.7275

**Table A9:** Optimal sampling termination thresholds for different datasets.

Dataset Name	Optimal Sampling Termination Threshold
CiteSeer	0.03
CiteSeer-full	0.02
Cora	0.05
Cora-ML	0.02
Facebook 107	0.1
Facebook 414	0.01/0.09
Facebook 1912	0.04
LFR-500	0.05
LFR-1000	0.02
